# MicroRNA-214 modulates the senescence of vascular smooth muscle cells in carotid artery stenosis

**DOI:** 10.1186/s10020-020-00167-1

**Published:** 2020-05-14

**Authors:** Yi-Ling Chen, Jiunn-Jye Sheu, Cheuk-Kwan Sun, Tien-Hung Huang, Yuan-Ping Lin, Hon-Kan Yip

**Affiliations:** 1 Division of Cardiology, Department of Internal Medicine, Kaohsiung Chang Gung Memorial Hospital and Chang Gung University College of Medicine, Kaohsiung, 83301 Taiwan; 2 Institute for Translational Research in Biomedicine, Kaohsiung Chang Gung Memorial Hospital, Kaohsiung, 83301 Taiwan; 3 Division of thoracic and Cardiovascular Surgery, Department of Surgery, Kaohsiung Chang Gung Memorial Hospital and Chang Gung University College of Medicine, Kaohsiung, 83301 Taiwan; 4 Center for Shockwave Medicine and Tissue Engineering, Kaohsiung Chang Gung Memorial Hospital, Kaohsiung, 83301 Taiwan; 5 Department of Emergency Medicine, E-Da Hospital, I-Shou University School of Medicine for International Students, Kaohsiung, 82445 Taiwan; 6 Department of health and Beauty, Shu-Zen Junior College of Medicine and Management, Kaohsiung, 82144 Taiwan; 7 Department of Medical Research, China Medical University Hospital, China Medical University, Taichung, 40402 Taiwan; 8 Department of Nursing, Asia University, Taichung, 41354 Taiwan; 9 Division of Cardiology, Department of Internal Medicine, Xiamen Chang Gung Hospital, Xiamen, 361028 Fujian China

**Keywords:** microRNA-214, Proliferation, Senescence, Vascular smooth muscle cells

## Abstract

**Background:**

MicroRNAs control gene expression by post-transcriptional inhibition. Dysregulation of the expressions of miR-199a/214 cluster has been linked to cardiovascular diseases. This study aimed at identifying potential microRNAs related to vascular senescence.

**Methods:**

Seven candidate microRNAs (miR-19a, −20a, −26b, −106b, − 126, − 214, and − 374) related to cell proliferation were tested for their expressions under CoCl_2_-induced hypoxia in vascular smooth muscle cells (VSMCs). After identification of miR-214 as the candidate microRNA, telomere integrity impairment and cell cycle arrest were examined in VSMCs by using miR-214 mimic, AntagomiR, and negative controls. To investigate the clinical significance of miR-214 in vascular diseases, its plasma level from patients with carotid artery stenosis (CAS) was assessed by quantitative reverse transcriptase polymerase chain reaction (qRT-PCR).

**Results:**

CoCl_2_ treatment for 48 h suppressed cell proliferation and angiogenesis as well as enhanced cell senescence in VSMCs. Besides, miR-214 level was elevated in both intracellular and exosome samples of VSMCs after CoCl_2_ treatment. Manipulating miR-214 in VSMCs demonstrated that miR-214 not only inhibited angiogenic and proliferative capacities but also promoted senescence through the suppression of quaking. Additionally, circulating miR-214 level was upregulated in CAS patients with high low-density lipoprotein cholesterol (LDL-C) value.

**Conclusion:**

Our findings suggested that miR-214 plays a role in the modulation of VSMC angiogenesis, proliferation, and senescence with its plasma level being increased in CAS patients with elevated LDL-C value, implying that it may be a vascular senescence marker and a potential therapeutic target for vascular diseases.

## Introduction

Despite therapeutic advances in cardiovascular disease, stroke remains the second leading cause of death worldwide as well as the third leading cause of disability and lost productivity (Mathers et al., 2017). There are two types of stroke, namely, ischemic stroke and hemorrhagic stroke. The former, which is attributed to a blood clot on an atherosclerotic plaque within a blood vessel in the brain that subsequently cuts off blood supply to that part of the brain, is the most common (Marulanda-Londono & Chaturvedi, 2016; Min et al., 1999; Rothwell, 2007). Vascular smooth muscle cells (VSMCs) play a pivotal role in atherogenesis. In particular, VSMC senescence in the fibrous cap is known to contribute to inefficient plaque repair that predisposes to subsequent plaque instability and rupture (Gorenne et al., 2006). However, the underlying genetic mechanisms of plaque rupture have not been fully elucidated.

MicroRNAs (miRNAs) are a family of small, noncoding RNAs, approximately 22 nucleotides in length involved in the regulation of gene expression at the post-transcriptional level by degrading their target mRNAs and/or inhibiting their translation (Bartel, 2004). As negative regulators of gene regulation, thousands of miRNAs contribute to various essential physiological and pathophysiological processes in human (Cui et al., 2012; Liao et al., 2013). Therefore, it is not surprising that miRNAs are also involved in the pathogenesis of cardiovascular diseases because of their multiple biological functions (Chen et al., 2010; Dimmeler & Nicotera, 2013; Harries, 2014; Jung & Suh, 2014; Lee et al., 2015; Liu et al., 2012). Recently, increasing evidence has shown that the miRNAs expression profiles are dysregulated during cellular senescence in aging and age-related diseases. Some miRNAs target conserved pathways of senescence, including the insulin/insulin-like growth factor pathway, sirtuin regulatory pathway, and the mammalian target of rapamycin pathway. Several pathways were associated with senescence and cardiovascular diseases (Chen et al., 2010; Lee et al., 2015; Jung & Suh, 2012; Mimura et al., 2014; Weilner et al., 2015). Therefore, the present study was designed to identify miRNAs, if any, that are differentially expressed during vascular dysfunction.

## Materials and methods

### Patients and samples

Venous blood samples were collected from 16 CAS patients with normal LDL-C level (< 100 mg/dL, *n* = 9) and high LDL-C level (> 100 mg/dL, *n* = 8) at the Kaohsiung Chang Gung Memorial Hospital, between 2018 and 2019. Plasma samples were then stored at − 80 °C until further processing. Blood levels of total cholesterol, triglyceride, low density lipoprotein-cholesterol (LDL-C), high density lipoprotein-cholesterol (HDL), fasting glucose and HbA1C were determined. All methods and experiments were performed in accordance with the approved guidelines of Kaohsiung Chang Gung Memorial Hospital (201601805B0C501).

### Reagents and antibodies

Cobalt chloride (CoCl_2_) was obtained from Sigma-Aldrich. The following primary antibodies were used for western blotting as validated in previous studies. Endothelial nitric oxide synthase (eNOS, CST-9572) (Yin et al., 2019; Hsu et al., 2019; Huang et al., 2017), phosphor-retinoblastoma protein (pRB, CST-9308) (Bernal et al., 2018) and stromal cell-derived factor-1α (SDF-1α, CST-3740) (Yin et al., 2019; Hsu et al., 2019; Huang et al., 2017) were from Cell Signaling Technology (Danvers, MA, USA). β-actin antibody (MAb1501) was from Merk Millipore (Dallas, TX, USA). Vascular endothelial growth factor A (VEGFA, ab1316), C-X-C chemokine receptor type 4 (CXCR4, ab124824), and quaking (ab126742) were from Abcam (Cambridge, UK) (Yin et al., 2019; Hsu et al., 2019; Huang et al., 2017). Hypoxia-inducible factor 1(HIF-1α, NB100–105) (Kornberg et al., 2018), telomeric repeat binding factor 1 (TERF1, NB110–68281) (Jullien et al., 2013), and telomeric repeat binding factor 2 (TERF2, NB110–56506) (Bernal et al., 2018) antibodies were from Novus Biologicals (Littleton, CO, USA). p2^1CIP1^ (sc-817) (Coni et al., 2017) and p16^INK4^ (sc-468) (Anderson et al., 2018) and antibodies were from Santa Cruze Biotechnology (Santa Cruze, CA, USA). MicroRNA mimic and antagomiR for miR-214 were from Qiagen (Foster City, CA, USA).

### Cell culture

Rat aortic vascular smooth muscle cell (A7r5) was purchased from Bioresource Collection and Research Center (Hsinchu, Taiwan). A75r cells were cultured in Dulbecco’s modified Eagle’s medium supplemented with 10% fetal bovine serum (Thermo Fisher Scientific, Grand Island, NY, USA) in a humidified atmosphere containing 5% CO_2_ and 95% air at 37 °C. Following serum starvation (1% FBS), cells were treated with or without CoCl_2_ for 2 days. Overexpression of miR-214 mimic or miR-214 inhibitor experiments were performed for 2–3 days in cells, according to the manufacturer’s instruction (Mirus Bio, Madison, Wisconsin, USA). All treatments were carried out three independent experiments done in triplicate.

### Isolation of total RNA and real-time reverse transcription-PCR (qRT-PCR)

Exosomes were isolated using Total Exosome Isolation kit™ (cat. 4,484,450, Thermo Fisher Scientific) from plasma and cell culture media. RNA was extracted from exosomal pellets and cultured cells using the RNeasy Mini Kit (Qiagen) according to the manufacturers’ instructions. For cDNA, DNase-treated total RNA was reverse-transcribed with the High-Capacity cDNA Reverse Transcriptase Kit (Cat. 4,368,814, Thermo Fisher Scientific) to produce complementary DNA (cDNA). Reverse transcription-generated cDNA encoding the target genes was amplified and quantified by QuantiNova™ SYBR® Green PCR kit (Cat. 208,054, Qiagen) using the primer sets shown in Table 1. For miRNA, total RNA containing miRNA was reverse-transcribed with miScript II RT Kit (Cat. 218,161, Qiagen) and amplified and quantified by miR-214 primers (Cat. MS00031605, 5′-ACAGCAGGCACAGACAGGCAGU-3′) and QuantiTect SYBR Green PCR Kit (Cat. 218,073, Qiagen). Samples were amplified in triplicates and copy numbers were calculated according to their cycle of threshold values. Relative quantification was performed by comparing the ratios of the target cDNA CT to those of the respective control RNU6B or cel-miR39.

**Table 1:** Primers for the study

Gene^a^	Accession number	Primer sequence (5′ to 3′) Forward/reverse	Amplicon (bp)
NOS3	NM_021838.2	F: CTGCGGTGATGTCACTATGG R: AAATGTCCTCGTGGTAGCGT	140
CXCR4	NM_022205.3	F: ATCATCTCCAAGCTGTCACACTCC R: GTGATGGAGATCCACTTGTGCAC	197
CXCL12	NM_022177.3	F: GCTCTGCATCAGTGACGGTAAG R: TGGCGACATGGCTCTCAAA	77
TERF1	NM_001012464.1	F: TACCAAACTCAAGCCCCATC R: GCAGCAAACTCACATCGAAA	170
TERF2	NM_001108448.1	F: AGAAGAAAGCGAGTGGGTGA R: TTGTGAGTCCTGTGGCTCTG	178
TERT	NM_053423.1	F: AGTGGTGAACTTCCCTGTGG R: CAACCGCAAGACTGACAAGA	232
VEGFA	NM_031836.3	F: TCGAGGAAAGGGAAAGGGTCAA R: TTTGCAGGAACATTTACACGTCTGC	139

^a^*Rattus norvegicus* database; *bp* base pair *Nos3*, nitric oxide synthase 3, *Cxcr4* C-X-C chemokine receptor type 4, *Cxcl12* stromal cell-derived factor 1, *Terf* telomeric repeat-binding factor, *Tert* telomerase reverse transcriptase, *VEGFA* vascular endothelial growth factor A

### Cell proliferation assay

Cell proliferation assays were performed by using Cell Counting Kit-8 (Sigma-Alrich). A7r5 were plated in 96-well plates at 10,000 cells per well and cultured in the growth medium. At the indicated time points, the viable cell numbers in triplicate wells were measured as the absorbance (450 nm) of reduced WST-8.

### Senescence-associated β-galactosidase (SA-β-gal) staining

This assay was detected using the senescence detection kit (ab65351, Abcam) according to the manufacturer’s instructions and analyzed with an Olympus Imaging System.

### Western blot analysis

Equal amounts (40–60 μg) of protein extracts were loaded and separated by SDS-PAGE with 10–15% acrylamide gradients (Chen et al., 2018). After electrophoresis, the separated proteins were electrophoretically transferred to a polyvinylidene difluoride (PVDF) membrane (Amersham Biosciences). Nonspecific proteins were blocked by incubating the membrane in blocking buffer (5% nonfat dry milk in T-TBS containing 0.05% Tween 20) for one hour at room temperature. The membranes were incubated with the appropriate primary antibodies against β-actin (1:3000), CXCR4 (1:1000), eNOS (1:1000), HIF-1α (1:1000), TERF1 (1:2000), TERF2 (1:2000), p16INK4 (1:1000), p21CIP1 (1:200), pRB (1:200), VEGF (1:1000) overnight. Horseradish peroxidase-conjugated anti-rabbit or anti-mouse immunoglobulin IgG (1:2000, Cell Signaling) was used as a second antibody for one hour at room temperature.

### Statistical analysis

All statistical analyses were performed using the SASS statistics software for Windows version 8.2 (SAS Institute, Cary, NC) package. Values were expressed as the means ± SEM. The Mann–Whitney test was performed to compare the differences in the plasma miRNA expression levels between the high LDL-C level patients and normal LDL-C level patients. The unpaired Student’s t-test was used to compare the differences in two independent samples. ANOVA followed by the Tukey-Kramer adjustment for multiple comparisons were used to evaluate differences among more than two groups.

## Results

### Effect of hypoxia on angiogenesis in VSMCs

**Fig. 1:**
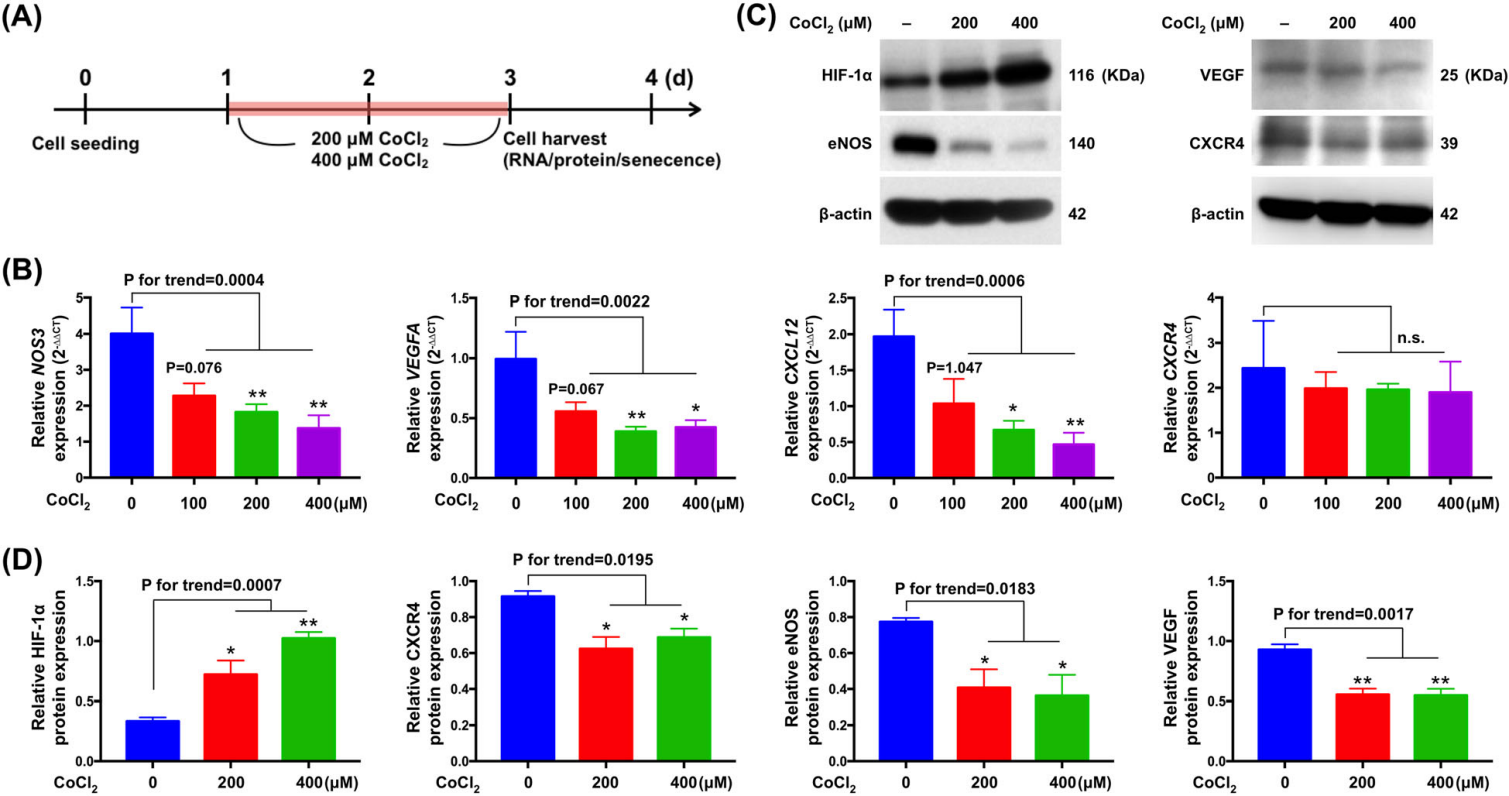
Cobalt (II) chloride (CoCl_2_) in rat vascular smooth muscle cells (A7r5) inhibited angiogenesis. **a** Schematic of the protocol for treatment of A7r5 VSMCs with CoCl_2_ or medium only. **b** mRNA expressions of NOS3, VEGFA, CXCL12 and CXCR4 compared in cells treated with CoCl_2_ or medium only (*n* = 3). **c** Representative western blots depicting HIF-1α, eNOS, VEGF and CXCR4 expression in CoCl_2_ and medium treated cells. **d** Normalized expressions of HIF-1α, eNOS, VEGF and CXCR4 (*n* = 3). Data are presented as the means ± SEM of three independent experiments performed by triplicate, * *P* < 0.05; ** *P* < 0.01; ns, non-significance (one-way ANOVA with Tukey-Kramer post hoc test)

To investigate the effect of hypoxia-induced oxidative stress (CoCl_2_, a commonly used hypoxia-mimetic agent) on angiogenesis in A7r5 VSMCs. A7r5 VSMCs were exposed to different concentrations of CoCl_2_ (100 μM, 200 μM, 400 μM) for 48 h in DMEM medium and expressions of angiogenesis-associated molecules were analyzed by qRT-PCR and western blotting (Fig. 1a). As shown in Fig. 1b, exposure to hypoxia at 200 μM and 400 μM CoCl_2_ for 48 h obviously reduced expressions of angiogenesis transcripts (i.e., NOS3, VEGFA, CXCL12). However, hypoxia at 100 μM CoCl_2_ had no obvious effect on VSMC angiogenesis. We then used 200 μM and 400 μM CoCl_2_ treatments as hypoxic stimuli in the following experiments. Under these hypoxic conditions, HIF-1α protein expression was remarkably increased in CoCl_2_-treated A7r5 VSMC when compared to that in controls, whereas expressions of angiogenesis proteins (i.e., eNOS, VEGF, CXCR4) were significantly decreased in CoCl_2_-treated A7r5 VSMCs (Fig. 1c-d). These data demonstrated that hypoxia at 200 μM and 400 μM CoCl_2_ suppressed VSMC angiogenesis in a dose-dependent manner.

### Impact of hypoxia on senescence in VSMCs

**Fig. 2:**
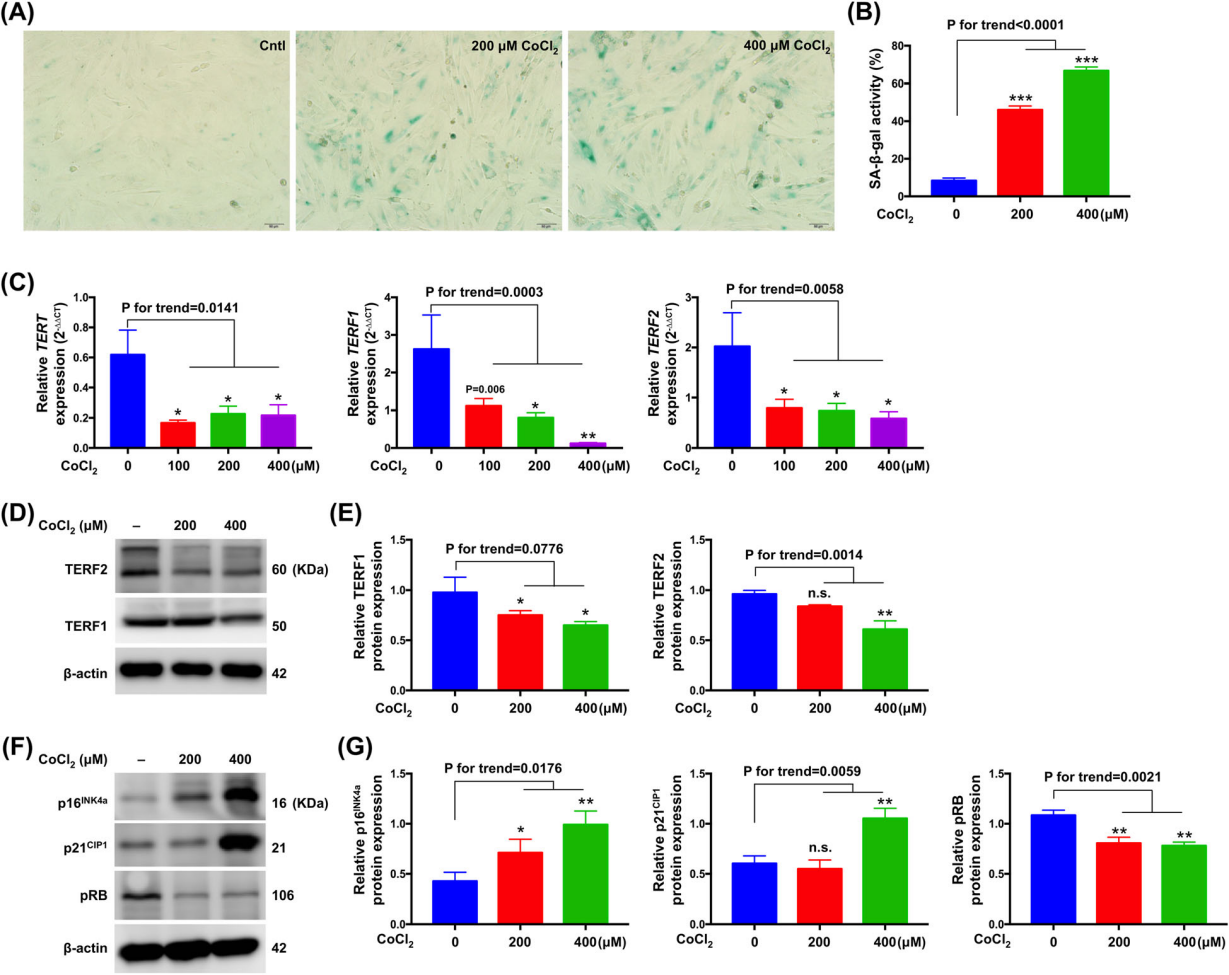
CoCl_2_ in rat vascular smooth muscle cells (A7r5) inhibited proliferation and induces senescence. **a** Senescence-associated-β-galactosidase staining demonstrating senescence in CoCl_2_ and medium treated cells. **b** Bar graphs show quantification of relative of SA-β-gal positive cells (*n* = 3). **c** mRNA expressions of TERT, TERF1 and TERF2 compared in cells treated with CoCl_2_ or medium only (n = 3). **d**, **f** Representative western blots depicting TERF1, TERF2, p16^INK4^, p21^CIP1^, pRB expression in CoCl_2_ and medium treated cells. **e**, **g** Normalized expressions of TERF1, TERF2, p16^INK4^, p21^CIP1^, pRB (*n* = 3). Data are presented as the means ± SEM.* *P* < 0.05; ** *P* < 0.01; *** *P* < 0.001; ns, non-significance (one-way ANOVA with Tukey-Kramer post hoc test)

To determine the effect of hypoxia-induced oxidative stress on expressions of hallmarks of senescence, A7r5 VSMCs were exposed to the same conditions as those in the experimental group. Cellular senescence was detected at various treatment concentrations with the commonly used marker SA-β-galactosidase. In contrast to spontaneous senescence that occurred only in a minority of A7r5 VSMCs with SA-β-galactosidase activity in normal controls, SA-β-galactosidase staining results showed that incubating in 200 μM CoCl_2_ for 48 h effectively induced senescence with a characteristic senescent phenotype. Moreover, the percentages of senescent cells increased with the treatment dosages (Fig. 2a-b). Furthermore, the results indicated that CoCl_2_ treatment downregulated molecular expressions of anti-senescence markers, including those of TERT, TERF1 and TERF2 transcripts and TERF1 and TERF2 proteins (Fig. 2c-e). Since previous evidence demonstrated that senescence-inducing signals usually involve either the p53 or the p16^INK4^-RB tumor suppressor pathways, we further investigated the expressions of cell cycle regulators (i.e., p16^INK4^, p21^CIP1^, pRB) after CoCl_2_ treatment. Western blot analyses showed that protein expressions of p16^INK4^ and p21^CIP1^ increased, whilst that of pRB deceased with increasing CoCl_2_ concentrations (Fig. 2f-g).

### Effects of hypoxia on cellular and exosomal levels of miR-214 in VSMCs

**Fig. 3:**
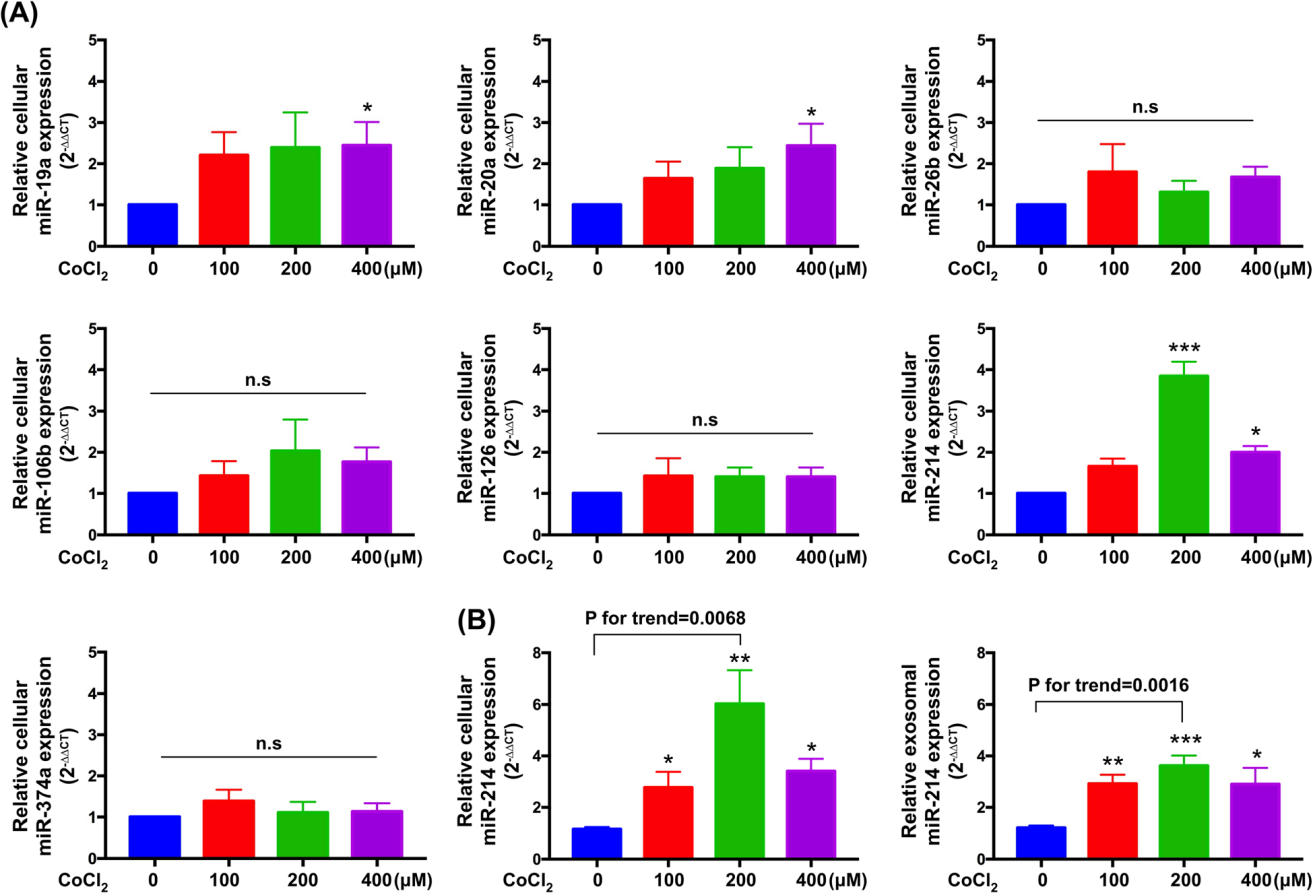
Cellular and exosomal microRNA-214-3p levels (miR-214) in response to CoCl_2_. **a** Mean normalized expression levels of A7r5 cellular miRNAs (miR-19a, −20a, −26b, −106b, − 126, − 214, and − 374) as determined by miRNA-specific SYBR Green PCR assays in response to CoCl_2_ for 48 h and normalized to RNU6B. Expression values are presented relative to medium only control (*n* = 6). **b** Total exosome isolation reagent isolated from A7r5 VSMCs after 48 h at CoC_2_ were spiked with cel miR-54 and used for RNA extractions. This exosomal RNA was then assayed for miR-214 and cel miR-39 by qRT-PCR and normalized to exogenous cel miR-54 (*n* = 6). Data are presented as the means ± SEM. * *P* < 0.05; ** *P* < 0.01; *** *P* < 0.001; ns, non-significance (one-way ANOVA with Tukey-Kramer post hoc test)

To identify the differentially expressed miRNAs in VSMCs with or without CoCl_2_ treatment, we detected the levels of seven candidate miRNAs (i.e., miR-19a, miR-20a, miR-26b, miR-106b, miR-126, miR-214, miR-374), which, evidence suggested, were related to cellular growth and survival (Lee et al., 2017; Meister & Schmidt, 2010; Penna et al., 2015). Of the three miRNAs showing significantly increased levels in A7r5 VSMCs after CoCl_2_ treatment on qRT-PCR analysis (i.e., miR-19a, miR-20a, and miR-214), miR-214 was the most highly expressed (Fig. 3a).

To evaluate the effects of hypoxia-induced oxidative stress on miR-214 cellular and exosomal levels, A7r5 VSMCs were stimulated for 48 h with different concentrations of CoCl_2_ (i.e., 0, 100 μM, 200 μM, 400 μM). At the end of treatment, cells and conditioned medium were collected and both total and exosomal RNAs were isolated. The results showed that, despite significant upregulation in cellular and exosomal miR-214 levels at CoCl_2_ concentrations of 100 μM, 200 μM, 400 μM, the most prominent increase in expression was noted at 200 μM (Fig. 3b).

### Role of miR-214 in regulating VSMC proliferation and senescence

**Fig. 4:**
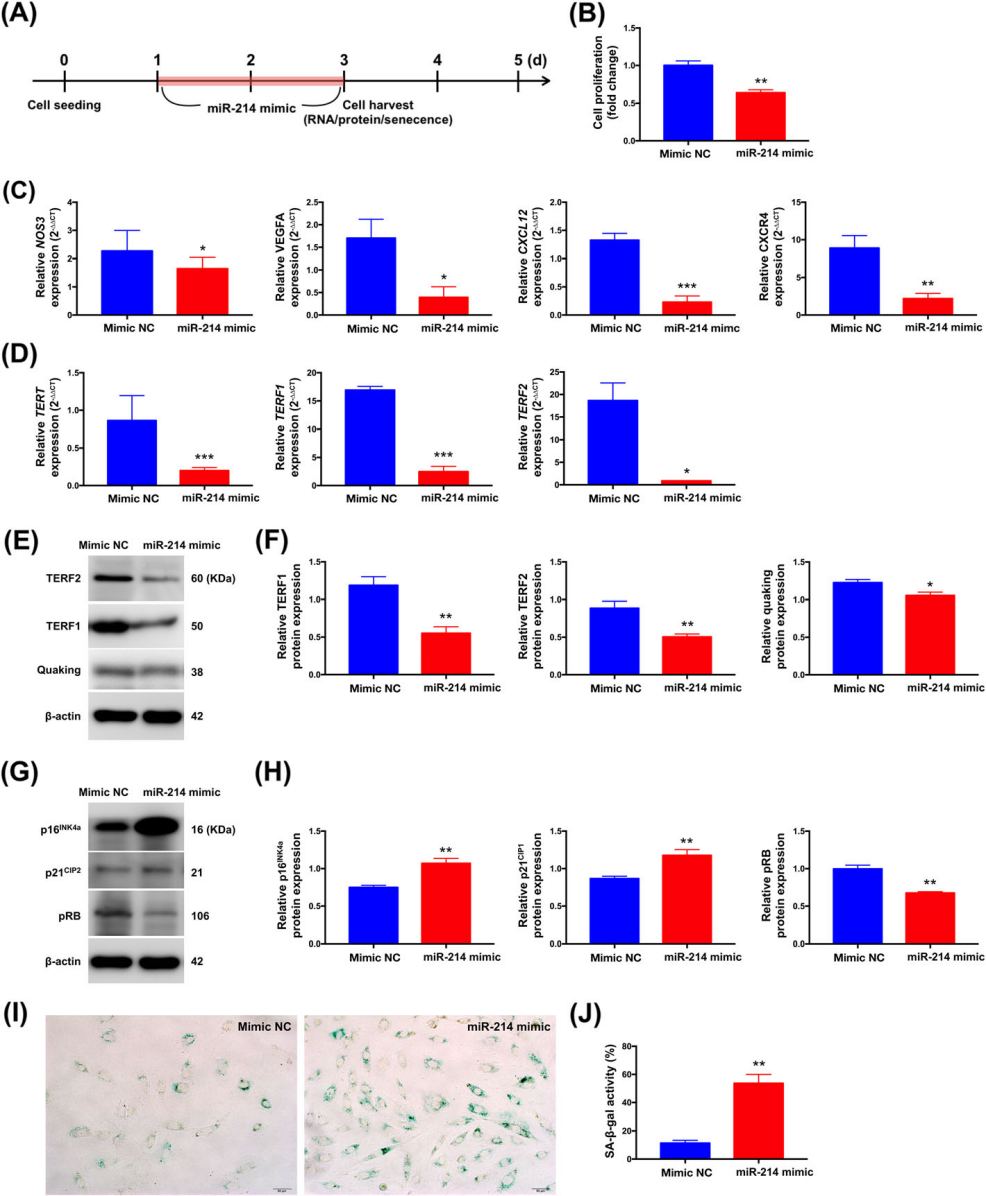
MicroRNA-214-3p (miR-214) mimic in rat vascular smooth muscle cells (A7r5) suppressed angiogenesis and proliferation but promoted senescence. **a** Schematic of the protocol for transfection of A7r5 VSMCs with a miRNA mimic control (mimic NC) or a miR-214 mimic and processed at the indicated times. **b** The effects of miR-214 mimic on VSMC cell proliferation were tested by CCK-8 assay. **c** Angiogenetic mRNA expressions of NOS3, VEGFA, CXCL12 and CXCR4 compared in cells transfected with mimic NC or miR-214 mimic (*n* = 3). **d** Senescence-associated mRNA expressions of TERT, TERF1 and TERF2 compared in cells transfected with mimic NC or miR-214 mimic (*n* = 3). **e**,**g** Representative western blots depicting TERF1, TERF2, p16^INK4^, p21^CIP1^, pRB, and quaking expression in mimic NC or miR-214 mimic transfected cells. **f**,**h** Normalized expressions of TERF1, TERF2, p16^INK4^, p21^CIP1^, pRB, and quaking (*n* = 3). **i** Senescence-associated β-galactosidase staining demonstrating senescence in mimic NC or miR-214 mimic transfected cells. **j** Bar graphs show quantification of relative of SA-β-gal positive cells (*n* = 3). Data are presented as the means ± SEM. * *P* < 0.05; ** *P* < 0.01; *** *P* < 0.001 (Two-tailed Student’s t-test)

To investigate whether miR-214 plays a role in VSMC growth/proliferation, we assessed the effects of upregulated and downregulated miR-214 expressions on VSMC growth/proliferation by transfecting A7r5 VSMCs with miR-214 mimic and antagomiR, respectively (Fig. 4a-b). The expressions of CXCL12, CXCR4, NOS3, VEGFA, TERT, TERF1, and TERF2 transcripts were reduced in miR-214-overexpressing A7r5 VSMCs (Fig. 4c-d). Further, miR-214 overexpression was associated with elevations in p16^INK4^, and p21^CIP1^ protein expressions but reductions in those of TERF1, TERF2, pRB, and quaking at 48 h post-transfection compared with control mimic (Fig. 4e-h). Accordingly, in comparison with control mimic, miR-214 overexpression also increased cellular SA-β-galactosidase activity (senescence) by 48 h after transfection (Fig. 4i-j).

**Fig. 5:**
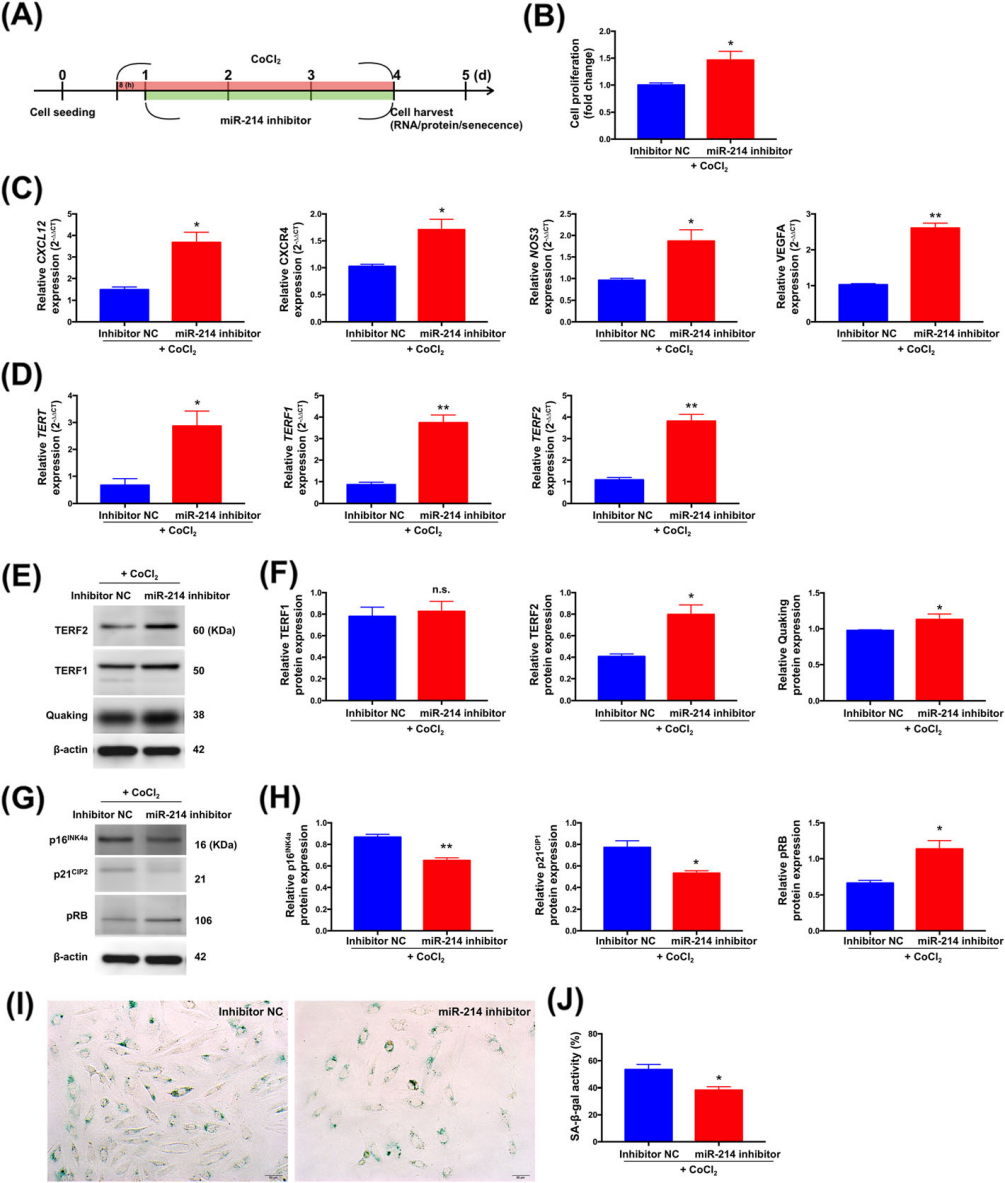
MicroRNA-214-3p (miR-214) antagomiR in rat vascular smooth muscle cells (A7r5) induced angiogenesis and proliferation but suppressed senescence. **a** Schematic of the protocol for transfection of A7r5 VSMCs with a miRNA antagomiR control (antagomiR NC) or a miR-214 antagomiR and processed at the indicated times. **b** The effects of miR-214 antagomiR on VSMC cell proliferation were tested by CCK-8 assay. **c** Angiogenetic mRNA expression levels of NOS3, VEGFA, CXCL12 and CXCR4 compared in cells transfected with antagomiR NC or miR-214 antagomiRs (*n* = 3). **d** Senescence-associated mRNA expression of TERT, TERF1 and TERF2 compared in cells transfected with antagomiR NC or miR-214 antagomiRs (*n* = 3). **e**, **G** Representative western blots depicting TERF1, TERF2, p16^INK4^, p21^CIP1^, pRB, and quaking expression in antagomiR NC or miR-214 antagomiRs transfected cells. **f**, **h** Normalized expression of TERF1, TERF2, p16^INK4^, p21^CIP1^, pRB, and quaking (*n* = 3). **i** Senescence-associated β-galactosidase staining demonstrating senescence in SCR or miR-214 transfected cells. **j** Bar graphs show quantification of relative of SA-β-gal positive cells (*n* = 3). Data are presented as the means ± SEM. * *P* < 0.05; ** *P* < 0.01; ns, non-significance (Two-tailed Student’s t-test)

Eight hours after CoCl_2_ treatment, A7r5 VSMCs were transfected with a specific antisense inhibitor for miR-214 (i.e., miR-214 antagomiR) (Fig. 5a). Interestingly, compared with control antagomiR, transfection of miR-214 antagomiR induced increases in CXCL12, CXCR4, NOS3, VEGFA, TERT, TERF1, and TERF2 transcript expressions and TERF1, TERF2, pRB, and quaking protein expressions in A7r5 VSMCs. However, we found significant reductions in p16^INK4^, and p21^CIP1^ protein expressions in A7r5 VSMCs overexpressing miR-214 antagomiR (Fig. 5c-h). We also observed that miR-214 antagomiR promoted VSMC proliferation (Fig. 5b) and suppressed VSMC senescence following CoCl_2_ treatment (Fig. 5i-j). Consistently, similar effects were also observed in human aortic smooth muscle cells (HASMCs) transfected with miR-214 mimic or miR-214 antagomiR (supplemental Fig. 1). Taken together, these findings showed that miR-214 could aggravate senescence of VSMCs under hypoxia.

### Upregulation of miR-214 in plasma of CAS patients with high LDL-C level

**Fig. 6:**
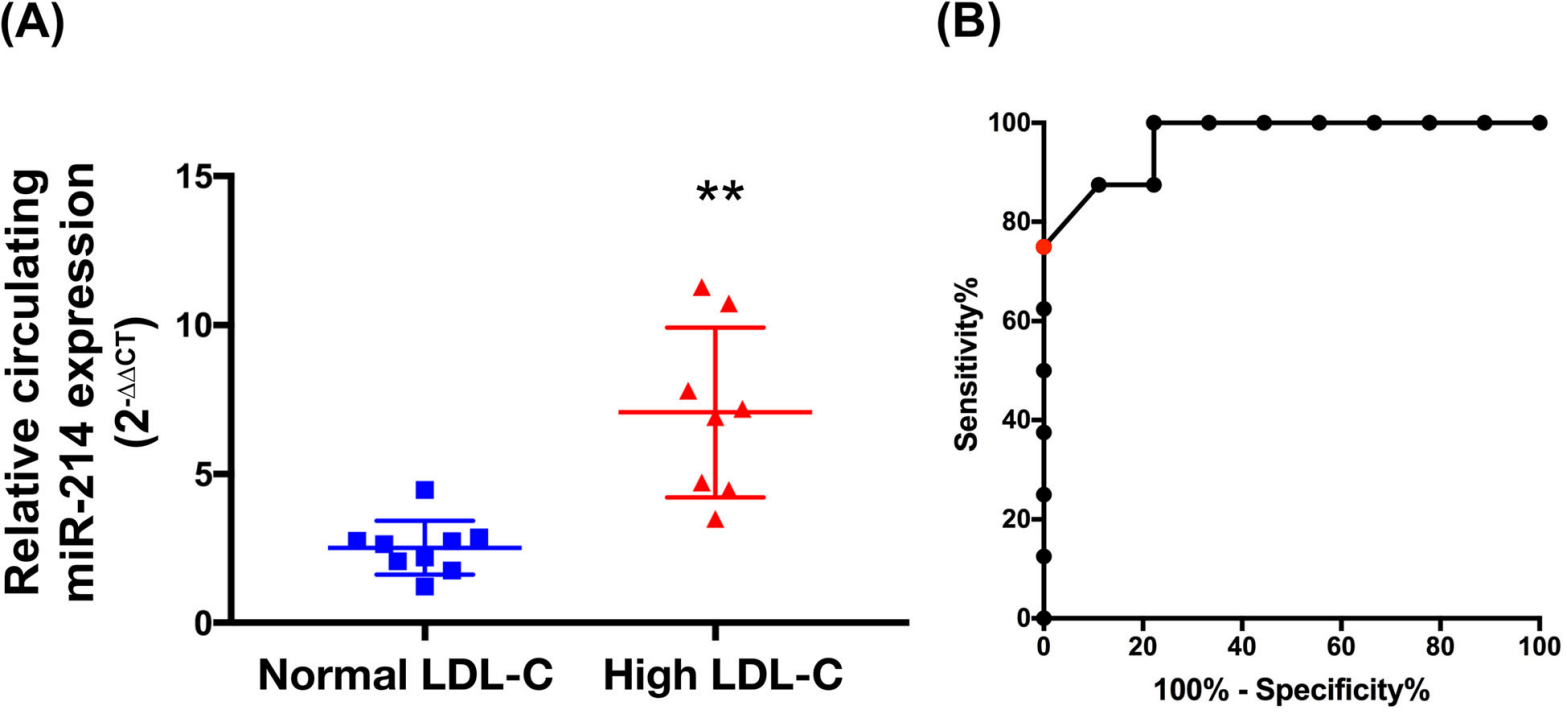
MicroRNA-214-3p (miR-214) was upregulated in plasma from carotid artery stenosis patients with elevated LDL-C value. **a** The expression of miR-214 in plasma of CAS patients with normal LDL-C value (*n* = 9) and CAS patients with high LDL-C value (*n* = 8) were analyzed by qRT-PCR and normalized to corresponding cel miR-39 levels (Two-tailed Student’s t-test). Data are presented as the means ± SEM. Differences were analyzed by Mann-Whitney test. ** *P* < 0.01 (Two-tailed Student’s t-test). **b** Assessment of the receiver operator characteristic (ROC) curve for the predictive power of the mir-214 circulating level in patients having high LDL-C level. The area under the ROC curve (AUC) was 0.96 (*p* = 0 0013). The optimal cut-off point is labeled by the red solid circle

To demonstrate whether miR-214 is dysregulated in CAS patients with elevated LDL-C level, we measured the plasma level of miR-214 in two groups of subjects. Baseline characteristics of these groups are displayed in the Table 2. One group comprised CAS patients diagnosed with high LDL-C level above 100 mg/dL (*n* = 8), whilst the other consisted of normal LDL-C level patients below 100 mg/dL (*n* = 9). qRT-PCR analysis revealed a 2.7-fold increase in subjects with elevated LDL-C level compared to that in that without (Fig. 6a). To clarify the power of mir-214 circulating level in predicting CAS outcomes of patients having high LDL-C level, receiver operator characteristic (ROC) curve analysis was performed (Fig. 6b). An optimal cut-off point at 3.12 was determined using the ROC curve. When patients were divided according to the cut-off point, sensitivity and specificity of the high score were 77.78 and 100%, respectively. Furthermore, if we want to correctly identify the high score of patients (more than 85%), an appropriate value of the cut-off point was considered to be 4.05 with sensitivity 87.5% and specificity 88.89%, respectively.

**Table 2:** Characteristics for the study population

Characteristics	High LDL-C case (*n* = 8) (> 100 mg/dL)	Low LDL-C case (*n* = 9) (< 100 mg/dL)	*P*-value
Age (years)	66.6 ± 7.3	72.0 ± 11	0.29
Male/female (n/n)	6/2	7/2	> 0.05
Diabetes mellitus, n (%)	1 (0.1%)	2 (0.2%)	0.63
Hypertension, n (%)	6 (0.8%)	8 (0.9%)	0.48
Total cholesterol	223.5 ± 52.7	129.0 ± 23.9	< 0.001
Triglyceride (mg/dL)	116.4 ± 40.4	101.4 ± 40.1	0.47
LDL- C (mg/dL)	148.8 ± 48.5	64.0 ± 17.4	< 0.001
HDL-C (mg/dL)	51.4 ± 8.8	41.1 ± 6.1	< 0.05
Fasting glucose (mmol/L)	110.9 ± 34.7	110.7 ± 23.3	0.99
HbA1c	6.5 ± 0.9	6.1 ± 0.8	0.33

Mean ± standard deviation. *LDL- C* low-density lipoprotein-cholesterol, *HDL-C* low-density lipoprotein-cholesterol. *P*, comparison between patients with low LDL-C

## Discussion

Several recent reports have shown that VSMC played a crucial role in atherosclerosis (Bennett et al., 2016; Chistiakov et al., 2015; Grootaert et al., 2018). Recently, increasing evidence has revealed that miRNAs are involved in atherosclerosis through the regulation of VSMC apoptosis and senescence (Clarke et al., 2006; Tan et al., 2016). To investigate the molecular mechanisms underlying VSMC senescence, we set out to identify miRNAs that regulated VSMC senescence in the present study and demonstrated that miR-214 promoted senescence of VSMCs. We found that overexpression and knockdown of miR-214 significantly promoted and inhibited the expressions of senescence genes (i.e., cell cycle regulators: p16^INK4^, p21^CIP1^ and pRB; telomere complex: TERT, TERF1 and TERF2) in A7r5 VSMCs, respectively. We also demonstrated that miR-214 in VSMCs modulated SA-β-galactosidase activity in vitro. Clinically, the plasma level of miR-214 was increased in CAS patients with elevated LDL-C level compared to those without in the present study. Our results indicated that miR-214 may perpetuate the senescence process through telomere integrity impairment and cell cycle arrest in VSMCs. Besides, our findings demonstrated that circulating miR-214 may serve as a promising biomarker for differentiating between CAS patients with/without elevated LDL-C level. The current study is the first investigation into the role of miR-214 in vascular senescence.

The miR-214 is highly conserved among species, suggesting its involvement in broad physiological and pathological functions. Interestingly, circulating miR-214 level has been shown to be of diagnostic potential for cardiovascular diseases. For instance, miR-214 was upregulated in the serum as well as in the hypertrophic and failing hearts of patients with heart failure (Bostjancic et al., 2009; Duan et al., 2015). Similarly, plasma miR-214 levels were significantly increased in CAD patients, thereby suppressing VEGF expression and EPC activities (Jin et al., 2015). Consistent with the findings of previous studies, we observed that circulating miR-214 level in CAS patients with high LDL-C value was significantly higher than that without. Therefore, these observations reinforce the potential of extracellular miR-214 use as a diagnostic or prognostic biomarker for cardiovascular diseases.

Increased VSMC proliferation is observed during early atherogenesis and upon vascular injury (Bennett et al., 2016; Lutgens et al., 1999). In contrast, VSMCs in advanced atherosclerotic plaques were characterized by numerous markers of senescence, including telomere shortening, expression of the cyclin-dependent kinase inhibitors p^16INK4^ and p21^CIP1^, and activity of senescence-associated β-galactosidase (Bennett et al., 1995; Matthews et al., 2006; O'Brien et al., 1993; Wang et al., 2015). In addition, VSMC senescence not only is an indicator of atherogenesis but also contributes directly to atherogenesis. Clinically, the most serious complication of atherosclerosis is plaque rupture that leads to myocardial infarction or stroke (Lusis, 2000). To date, there is no universal indicator for VSMC senescence that helps in early identification of stenotic vascular diseases and staging of their severity. The findings of the present study suggested that plasma miR-214 level may be a potential indicator and staging tool for stenotic vascular diseases.

Endothelial cells and VSMCs are the major cellular components of the vasculature (Li et al., 2018). So far, approximately 2200 miRNA genes have been identified in the mammalian genome, from which over 1000 belong to the human genome (Ardekani & Naeini, 2010). Many miRNAs participate in regulating the function of both endothelial cells and VSMCs, including differentiation, migration, proliferation, senescence, and apoptosis (Lin et al., 2016). Several previous studies have identified miR-214 as a negative regulator of angiogenesis through its ability of directly targeting pro-angiogenic genes (i.e., eNOS, XBP1, and Quaking) in endothelial cells (Duan et al., 2015; Chan et al., 2009; van Mil et al., 2012). In contrast to the known miR-214-targeting genes in angiogenesis, the corresponding genes associated with senescence for miR-214 have not been identified well. Among mentioned above genes for mir-214, we focused on Quaking for two reasons. First, miR-214 has been reported to mediate in VSMC differentiation from embryonic stem cells (ESC) by targeting Quaking. Quaking downregulated the VSMCs gene expression (i.e., SRF, MEF2C and Myocd) through a transcriptional mechanism during ESC-VSMC differentiation (Wu et al., 2017). Second, Quaking was also found to regulate senescence- associated secretory phenotype (SASP) genes and to maintain telomere function as well as to regulate cell proliferation (Chen et al., 2015; Novikov et al., 2011). On the other hand, we used quantitative real-time RT-PCR to demonstrate that cellular and exosomal levels of miR-214 were upregulated in human aortic smooth muscle cells (HASMCs) after treatment with CoCl_2_ (supplemental Fig. 1A), similar to those obtained from rat A7r5 cells (Fig. 3b). In supplemental Fig. 1, after transfection of miR-214 mimic in HASMCs, telomere-associated protein expressions were decreased, such as quaking, TERF1, TERF2, leading to cellular senescence; moreover, loss of pRB and gain of p16^INK4^ caused cell cycle dysregulation, resulting in inhibited proliferation (supplemental Fig. 1B-C, Fig. 1f-g, Fig. 1j-k). On the contrary, opposite effects were displayed in HASMCs after miR-214 antagomiR transfection and CoCl_2_ treatment (supplemental Fig. 1D-E, Fig. 1h-i, Fig. 1l-m). Therefore, all these findings suggest that miR-214 is an important regulator of VSMC senescence via regulating the quaking expression.

## Conclusion

**Fig. 7:**
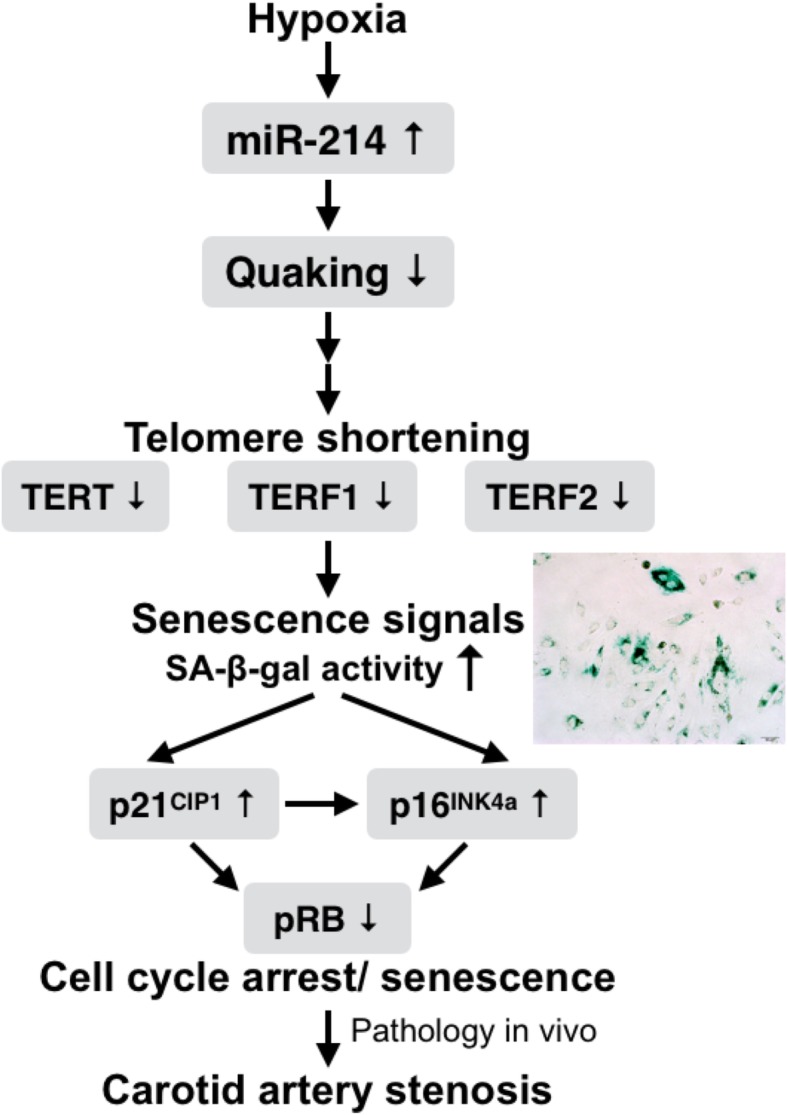
Schematic diagram represents the proposed mechanism of action of microRNA-214 (miR-214) in senescent VSMCs. In response to hypoxia-induced oxidative stress, hypoxia-induced microRNA-214 were overexpresed and promoted A7r5 VSMCs enter a senescent state. In senescent VSMCs, miR-214 targeted quaking to trigger loss of telomere integrity, upregulation of the cell cycle inhibitors p16^INK4^ and/or p21^CIP1^, and accumulation of senescence-associated β-galactosidase (SA-β-Gal). Therefore, miR-214 appeared to induce VSMC senescence and cell death, further jeopardize plaque stability in carotid artery stenosis. Arrows (—>) indicated molecular interaction or relation with miR-214

The findings of the present study opened up an avenue to further research on the role of non-coding RNAs in the vascular senescence process. In summary, this study suggested that hypoxia-induced miR-214 expression targeted quaking, which is associated with promoting VSMC senescence with telomere shortening and/or uncapping. Because cyclin dependent kinases are responsible for RB phosphorylation, the resulting increase in the expressions of cyclin dependent kinase inhibitors (i.e., p16^INK4^ and p21^CIP1^) leads to reduced pRB expression (Fig. 7).

### Limitations

This study has limitations. First, the limited number of patients in this study precluded investigation into the correlation between miR-214 expression levels and severity of carotid artery stenosis. Second, despite the identification of the role of miR-214 in senescence of VSMCs, the corresponding genes have not been identified. Third, the current study did not include rodent model of carotid artery stenosis to show the miR-214-mediated VSMC senescence in vivo. In conclusion, the results of the present study suggested that miR-214 may exacerbate vascular aging by promoting VSMC senescence both ex vivo and in vitro. Understanding the biology of miR-214 could potentially lead to the development of miR-214 as a possible biomarker and a therapeutic target for cardiovascular diseases.

## Supplementary information

**Additional file 1: Supplemental materials and methods. Figure S1.** MicroRNA-214-3p (miR-214) modulates the snenecence of human aortic smooth muscle cells induced by hypoxia. (A) The cellular and exosomal miR-214 in CoC_2_ treated HASMCs after 48 h (*n* = 5). (B,D) Representative western blots depicting quaking, TERF1, TERF2, p16^INK4^, and pRB expression in miR-214 mimic and miR-214 antagomiR transfected HASMCs (*n* = 3). (C,E) Normalized expressions of quaking, TERF1, TERF2, p16^INK4^, and pRB (*n* = 3). (F,H) Senescence-associated β-galactosidase staining demonstrating senescence in miR-214 mimic and miR-214 antagomiR transfected HASMCs. (G,I) Bar graphs show quantification of relative of SA-β-gal positive cells (*n* = 3). (J,L) Immunofluorescent staining showing the number of Ki-67 positive cells (pink; shown by white arrow) in miR-214 mimic and miR-214 antagomiR transfected HASMCs. (K,M) Bar graphs show quantification of relative of Ki-67 positive cells (*n* = 3). Bar, 100 μm. Data are presented as the means ± SEM. * *P* < 0.05; ** *P* < 0.01 (Two-tailed Student’s t-test).

## Data Availability

We do not wish to publicly share our data. Please contact us for data requests.

## Abbreviations

CAS: Carotid artery stenosis
CXCR4: C-X-C chemokine receptor type 4
CoCl_2_: Cobalt chloride
eNOS: cendothelial nitric oxide synthase
HIF-1α: Hypoxia-inducible factor 1
LDL-C: Low-density lipoprotein cholesterol
miRNAs: microRNAs
pRB: phosphor-retinoblastoma protein
qRT-PCR: quantitative reverse transcriptase polymerase chain reaction
SDF-1α: Stromal cell-derived factor-1α
TERFs: Telomeric repeat binding factors
SA-β-gal: Senescence-associated β-galactosidase
VSMCs: Vascular smooth muscle cells
VEGFA: Vascular endothelial growth factor A
