# Supplementary material for: MicroRNA-214 modulates the senescence of vascular smooth muscle cells in carotid artery stenosis
Source: Mol Med. 2020 May 14;26:46. doi: 10.1186/s10020-020-00167-1 (PMC7227274; doi:10.1186/s10020-020-00167-1)
Supplement: Supplementary file 1 — Additional file 1: Supplemental materials and methods. Figure S1. MicroRNA-214-3p (miR-214) modulates the snenecence of human aortic smooth muscle cells induced by hypoxia. (A) The cellular and exosomal miR-214 in CoC2 treated HASMCs after 48 h (n = 5). (B,D) Representative western blots depicting quaking, TERF1, TERF2, p16INK4, and pRB expression in miR-214 mimic and miR-214 antagomiR transfected HASMCs (n = 3). (C,E) Normalized expressions of quaking, TERF1, TERF2, p16INK4, and pRB (n = 3). (F,H) Senescence-associated β-galactosidase staining demonstrating senescence in miR-214 mimic and miR-214 antagomiR transfected HASMCs. (G,I) Bar graphs show quantification of relative of SA-β-gal positive cells (n = 3). (J,L) Immunofluorescent staining showing the number of Ki-67 positive cells (pink; shown by white arrow) in miR-214 mimic and miR-214 antagomiR transfected HASMCs. (K,M) Bar graphs show quantification of relative of Ki-67 positive cells (n = 3). Bar, 100 μm. Data are presented as the means ± SEM. * P < 0.05; ** P < 0.01 (Two-tailed Student’s t-test). [file 10020_2020_167_MOESM1_ESM.zip › 10020_2020_167_MOESM1_ESM/Supplemental materials and methods.docx]

**Supplements**

**Supplemental materials and methods**

**Cell culture**

Human aortic smooth muscle cells (HASMCs), purchased from ScienCell (Carlsbad, CA, USA), were cultured in Smooth Muscle Cell Medium (Cat.1101, ScienCell).

**Immunofluorescent (IF) staining**

Briefly, HASMCs were fixed with 4% paraformaldehyde followed by permeabilization with 0.1% Triton X-100. Fixed and permeabilized cells were labeled were incubated with diluted murine anti-Ki-67 monoclonal antibody (1:500, ab155801, Abcam) and diluted goat-anti-rabbit Alexa Fluor-conjugated antibody (1:200, cat. A-11037, ThermoFisher), as well as counterstained with DAPI. The cells were examined by fluorescence microscopy (Olympus)

**Supplemental Figure 1. MicroRNA-214-3p (miR-214) modulates the senescence of human aortic smooth muscle cells induced by hypoxia.** (A) The cellular and exosomal miR-214 in CoC_2_ treated HASMCs after 48 hours (n=5). (B,D) Representative western blots depicting quaking, TERF1, TERF2, p16^INK4^, and pRB expression in miR-214 mimic and miR-214 antagomiR transfected HASMCs (n=3). (C,E) Normalized expressions of quaking, TERF1, TERF2, p16^INK4^, and pRB (n=3). (F,H) Senescence-associated β-galactosidase staining demonstrating senescence in miR-214 mimic and miR-214 antagomiR transfected HASMCs. (G,I) Bar graphs show quantification of relative of SA-β-gal positive cells (n=3). (J,L) Immunofluorescent staining showing the number of Ki-67 positive cells (pink; shown by white arrow) in miR-214 mimic and miR-214 antagomiR transfected HASMCs. (K,M) Bar graphs show quantification of relative of Ki-67 positive cells (n=3). Bar, 100 μm. Data are presented as the means ± SEM. * P< 0.05; ** P< 0.01 (Two-tailed Student’s t-test).
